# Risk Stratification for Imminent Risk of Death at the Time of Palliative Radiotherapy Consultation

**DOI:** 10.1001/jamanetworkopen.2021.15641

**Published:** 2021-07-01

**Authors:** Susan Y. Wu, Emily Yee, Harish N. Vasudevan, Shannon E. Fogh, Lauren Boreta, Steve E. Braunstein, Julian C. Hong

**Affiliations:** 1 Division of Radiation Oncology, University of Texas MD Anderson Cancer Center, Houston; 2 Department of Radiation Oncology, University of California, San Francisco

## Abstract

This cohort study of patients with advanced cancer who received palliative radiotherapy within 30 days of death assesses models of prognostic criteria for providing radiotherapy at the end of life and compares outcomes with similar prognostic tools.

## Introduction

Palliative radiotherapy (RT) is frequently an effective treatment option for patients with advanced cancer; however, a substantial proportion will receive RT at the end of life.^1^ Prognostic tools, including the TEACHH (Type of cancer, ECOG [Eastern Cooperative Oncology Group] performance status, Age, prior palliative Chemotherapy, prior Hospitalizations, and Hepatic metastases),^2^ the model developed by Chow et al,^3^ and NEAT (Number of active tumors, ECOG performance status, Albumin, primary Tumor)^4^ models, can help guide decision-making to better align treatment intensity and prognosis. However, these may not generalize to all clinical scenarios, particularly for patients at imminent risk of death.^5^ Given this important unmet clinical need, we sought to identify prognostic criteria for 30-day mortality among patients with metastatic cancer undergoing palliative RT at our institution.

## Methods

We performed a single-institution retrospective cohort study of 518 patients who received RT to a site of metastatic disease between 2012 and 2016. This cohort has been previously described^5^—patient characteristics and survival rates based on TEACHH,^2^ Chow,^3^ and NEAT^4^ scores are available in the eTable in the Supplement. Components of the TEACHH, Chow, and NEAT models, as well as additional clinical and RT-related variables, were obtained from the medical record. Vital status was confirmed by the tumor registry and public records. This study was approved by the institutional review board of the University of California, San Francisco, and informed consent was waived for being secondary research of a large number of patients, many of whom are no longer being followed at the institution or are deceased and for whom risk of contact would pose a greater risk than the study. This analysis followed the Strengthening the Reporting of Observational Studies in Epidemiology (STROBE) reporting guideline.

Statistical analysis was performed in R version 3.6.0 (R Project for Statistical Computing), with source code available in the eAppendix in the Supplement. All tests were 2-sided, with *P* < .05 considered statistically significant. Associations between clinical characteristics at the time of RT consultation and survival from the start of RT were assessed using the Cox proportional hazards method. We used a binary classification tree approach with recursive partitioning analysis to stratify patients into pretreatment prognostic classes for mortality within 30 days of RT, with a surrogate split for missing data.

## Results

We included 518 patients, with median (interquartile range [IQR]) age at RT of 63 (54-71) years; the cohort included 238 [45.9%] women, and 340 [65.6%] White, 74 [14.3%] East Asian, and 45 [8.7%] Black patients.^5^ The rate of mortality within 30 days of RT was 24.1% (125 of 518 patients). On multivariate analysis, breast or prostate primary tumor (hazard ratio [HR], 1.41; 95% CI, 1.02-1.95; *P* = .04), ECOG score (HR, 1.18; 95% CI, 1.00-1.38; *P* = .048), body mass index (calculated as weight in kilograms divided by height in meters squared) (HR, 0.977; 95% CI, 0.956-0.999; *P* = .04), liver metastases (HR, 1.43; 95% CI, 1.09-1.89; *P* = .01), more than 5 active metastases (dichotomized, radiographically identified) (HR, 1.63; 95% CI, 1.04-2.57; *P* = .04), albumin level (HR, 0.78; 95% CI, 0.62-0.99; *P* = .04), and hospitalization within 3 months of RT consult (HR, 1.88; 95% CI, 1.38-2.55; *P* < .001) were associated with survival from the start of RT (Table).

**Table.** Univariate and Multivariate Analysis of Survival Following Palliative RT

Characteristic	Univariate analysis, HR (95% CI)	*P* value^a^	Multivariate analysis, HR (95% CI)	*P* value^a^
Age at RT	1.00 (0.99-1.01)	.69	NA	NA
ECOG score	1.46 (1.32-1.62)	<.001	1.18 (1.00-1.38)	.05
Breast or prostate primary tumor	1.50 (1.23-1.82)	<.001	1.41 (1.02-1.95)	.04
Metastases				
Liver	1.45 (1.21-1.73)	<.001	1.43 (1.09-1.89)	.01
Brain	1.35 (1.13-1.61)	<.001	1.26 (0.96-1.65)	.09
Lung	1.23 (1.03-1.47)	.02	1.29 (0.97-1.70)	.08
>5 Active metastases	1.55 (1.12-2.15)	.008	1.63 (1.04-2.57)	.04
Albumin level	0.61 (0.50-0.84)	<.001	0.78 (0.62-0.99)	.04
Hospitalization within 3 mo	1.87 (1.56-2.25)	<.001	1.88 (1.38-2.55)	<.001
BMI	0.98 (0.96-0.99)	.004	0.98 (0.96-1.00)	.04
Prior palliative chemotherapy regimens	0.93 (0.89-0.97)	.001	0.95 (0.89-1.02)	.17
Metastatic at diagnosis	1.07 (0.90-1.28)	.44	NA	NA

Abbreviations: BMI, body mass index; ECOG, Eastern Cooperative Oncology Group; HR, hazard ratio; NA, not applicable; RT, radiotherapy.

^a^ Statistical significance set at *P* < .05.

Recursive partitioning analysis of patients for 30-day mortality identified 5 classes (Figure): class 1, outpatients with an ECOG score below 3 (30-day mortality, 13%; 95% CI 8%-18%); class 2, outpatients with an ECOG score 3 or above without liver metastases (14%; 95% CI, 7%-21%); class 3, outpatients with an ECOG score of 3 or above with liver metastases (30%; 95% CI, 16%-41%); class 4, inpatients younger than 68 years (34%; 95% CI, 25%-42%); and class 5, inpatients 68 years of age or older (59%; 95% CI, 42%-70%).

**Figure.**
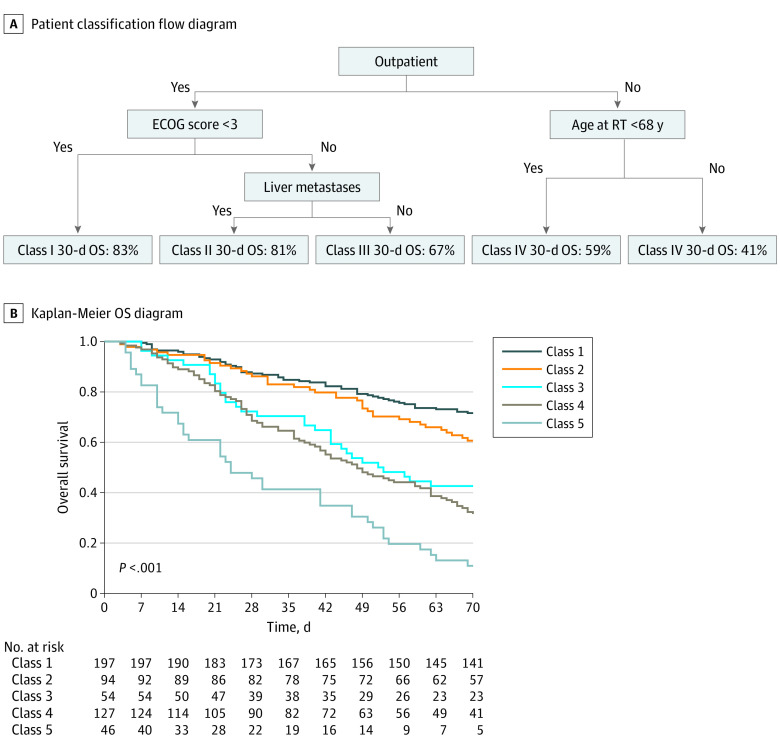
Recursive Partitioning Model for 30-Day Mortality ECOG indicates Eastern Cooperative Oncology Group; OS, overall survival; and RT, palliative radiotherapy.

Compared with cohorts from models in previous studies, patients in our cohort may have had more advanced disease (eg, median [IQR] time from diagnosis to RT, 28 [11-53] months vs TEACHH median [range] time from initial diagnosis to metastatic diagnosis, 0.3 [0-380] months, and from metastatic diagnosis to palliative RT consultation, 1.5 [0-274] months) and received more systemic therapy (patients receiving more than 2 courses of palliative chemotherapy: 43.1% [203 of 471 patients with available data] vs TEACHH, 17.1% [147 of 862 patients]). Although many patients received prior palliative RT (224 of 518 patients [43.2%] vs 12% [106 of 862 patients] in TEACHH), there was no difference in survival from RT for patients assessed at their first course compared with a subsequent course.

## Discussion

In this cohort study, we developed a classification system identifying 5 prognostic groups of patients undergoing palliative RT, with stratified 30-day mortality ranging from 13% to 59%. This used variables found across multiple survival models, highlighting the complexity of prognostication and the importance of applying models in appropriate clinical contexts. Our patients differed from those in prior models in several key ways, with more systemic treatment prior to palliative RT and potentially more advanced disease. The poor survival in our cohort may also have been influenced by the use of diagnostic codes to identify patients with secondary malignant neoplasms.

Our classification approach has the advantage of identifying interactions between clinical variables and suggests that performance status may be more relevant in the outpatient setting, while age may be more important for inpatients. Recursive partitioning analysis is also simple to interpret and apply clinically. More complex models developed using machine learning algorithms can be more nuanced, but also have limitations in their predictive ability.^6^

We used a recursive partitioning approach to develop a simple point of care tool that can be used to personalize treatment recommendations at the time of palliative RT consultation. Our study is limited by its retrospective nature and sample size, which limited our ability to detect further interactions between groups. Next steps include external validation and prospective application in a clinical setting.

## Notes

Supplement. **eTable.** Patient Characteristics **eAppendix.** Source Code
