# Supplementary material for: Risk Stratification for Imminent Risk of Death at the Time of Palliative Radiotherapy Consultation
Source: JAMA Netw Open. 2021 Jul 1;4(7):e2115641. doi: 10.1001/jamanetworkopen.2021.15641 (PMC8251501; doi:10.1001/jamanetworkopen.2021.15641)
Supplement: Supplement. — eTable. Patient Characteristics eAppendix. Source Code [file jamanetwopen-e2115641-s001.pdf]

Supplemental Online Content

Wu SY, Yee E, Vasudevan HN, et al. Risk stratification for imminent risk of death at the time of palliative radiotherapy consultation. *JAMA Netw Open.* 2021;4(7):e2115641. doi:10.1001/jamanetworkopen.2021.15641

eTable. Patient Characteristics

eAppendix. Source Code

This supplemental material has been provided by the authors to give readers additional information about their work.

© 2021 Wu SY et al. *JAMA Network Open.*

eTable. Patient characteristics

**Variable**	**Median (IQR) or % (n, of 518)**
Age at diagnosis	60 (50-68)
Age at palliative radiotherapy (RT)	63 (54-71)
Gender	
Female	46% (238)
Male	54% (280)
Race	
White	66% (340)
East Asian	14% (74)
African American	8.7% (45)
South Asian	7.3% (38)
Other (includes American Indian, Pacific Islander)	4.1% (21)
Percent Hispanic	9.1% (47)
Primary diagnosis	
Lung	26% (137)
Breast	19% (97)
Prostate	9.7% (50)
Renal cell	5.8% (30)
Colorectal	5.8% (30)
Hepatocellular	3.8% (20)
Head and Neck	3.5% (18)
Skin	3.3% (17)
Other*	23% (119)
Metastatic at diagnosis	49% (254)
Site of metastases	
Brain	47% (244)
Lung	55% (284)
Liver	40% (208)
Bone only	17% (90)
Body mass index last course	24 (21-27)
KPS last consult	60 (50-80)
KPS >70	45% (231/511)
Hospitalization within 3 months of RT consult	58% (289/500)
Survival time since diagnosis (months)	28 (11-53)
Survival time following RT (months)	2.2 (0.9-5.5)

© 2021 Wu SY et al. *JAMA Network Open.*

TEACHH score[2]	% (N*)	**Median survival in our cohort, (95% CI)**	**Published median survival, (95% CI, if available)**
0-1 (Group A)	6.2% (32/450)	6.0 mo (3.2-8.2)	20 mo (14-31)
2-4 (Group B)	68% (352/450)	2.0 mo (1.7-2.3)	5.0 mo (4.3-5.6)
5-6 (Group C)	13% (66/450)	1.3 mo (0.7-1.7)	1.7 mo (1.2-2.1)
Chow group[3]			
I	18% (92/510)	4.7 wks (2-11)	60 wks (37-70)†
II	44% (227/510)	2.5 wks (1.0-5.6)	26 wks (20-31)†
III	37% (191/510)	1.6 wks (0.7-2.7)	9.0 wks (6-11) †
**NEAT group (score)[4]**			
Very low risk (0-1)	19% (57/297)	5.4 mo	25 mo
Low risk (2)	29% (85/297)	3.3 mo	15 mo
Intermediate risk (2.5-3.5)	38% (114/297)	2.0 mo	4.0 mo
High risk (4-5)	14% (41/297)	1.4 mo	1.2mo

Abbreviations: RT: radiotherapy; KPS: Karnofsky performance status
*Denominator reflects limited data, scores only calculated if all variables available
†Training set, with similar numbers for the temporal and external validation sets

© 2021 Wu SY et al. *JAMA Network Open.*

eAppendix. Source Code

```
#univariate models for survival
require(survival)

agecox <- coxph(Surv(StartToDeath, rep(1, nrow(Data10092019)))~ AgeTx, data =
Data10092019)
summary(agecox)
cox.zph(agecox)#test proportional hazards assumption

agedxcox <- coxph(Surv(StartToDeath, rep(1, nrow(Data10092019)))~ AgeDx, data =
Data10092019)
summary(agedxcox)
cox.zph(agedxecox)#test proportional hazards assumption

racecox <- coxph(Surv(StartToDeath, rep(1, nrow(Data10092019)))~ RaceCod, data =
Data10092019)
summary(racecox)
cox.zph(racecox)#test proportional hazards assumption

txsitecox <- coxph(Surv(StartToDeath, rep(1, nrow(Data10092019)))~ TxSiteGen, data =
Data10092019)
summary(txsitecox)
cox.zph(txsitecox)#test proportional hazards assumption

codedsitecox <- coxph(Surv(StartToDeath, rep(1, nrow(Data10092019)))~ CodedBreastProstate,
data = Data10092019)
summary(codedsitecox)
cox.zph(codedsitecox)#test proportional hazards assumption

numpallchemocox <- coxph(Surv(StartToDeath, rep(1, nrow(Data10092019)))~
NumberPalliativeChemo, data = Data10092019)
summary(numpallchemocox)
cox.zph(numpallchemocox)#test proportional hazards assumption

bmicox <- coxph(Surv(StartToDeath, rep(1, nrow(Data10092019)))~ BMI, data = Data10092019)
summary(bmicox)
cox.zph(bmicox)#test proportional hazards assumption

hosp3cox <- coxph(Surv(StartToDeath, rep(1, nrow(Data10092019)))~ Hosp3moconsult, data =
Data10092019)
summary(hosp3cox)
cox.zph(hosp3cox)#test proportional hazards assumption
```

© 2021 Wu SY et al. *JAMA Network Open.*

```
hepcox <- coxph(Surv(StartToDeath, rep(1, nrow(Data10092019)))~ HepaticMet, data =
Data10092019)
summary(hepcox)
cox.zph(hepcox)#test proportional hazards assumption

ecogcox <- coxph(Surv(StartToDeath, rep(1, nrow(Data10092019)))~ ECOGscore, data =
Data10092019)
summary(ecogcox)
cox.zph(ecogcox)#test proportional hazards assumption

metdxcox <- coxph(Surv(StartToDeath, rep(1, nrow(Data10092019)))~ MetDx, data =
Data10092019)
summary(metdxcox)
cox.zph(metdxcox)#test proportional hazards assumption

braincox <- coxph(Surv(StartToDeath, rep(1, nrow(Data10092019)))~ BrainMet, data =
Data10092019)
summary(braincox)
cox.zph(braincox)#test proportional hazards assumption

lungcox <- coxph(Surv(StartToDeath, rep(1, nrow(Data10092019)))~ LungMet, data =
Data10092019)
summary(lungcox)
cox.zph(lungcox)#test proportional hazards assumption

numbmetcox <- coxph(Surv(StartToDeath, rep(1, nrow(Data10092019)))~ NumMets, data =
Data10092019)
summary(numbmetcox)
cox.zph(numbmetcox)#test proportional hazards assumption

albcox <- coxph(Surv(StartToDeath, rep(1, nrow(Data10092019)))~ Albuming, data =
Data10092019)
summary(albcox)
cox.zph(albcox)#test proportional hazards assumption


#multivariate model based on univariate and clinical judgment
mvcox <- coxph(Surv(StartToDeath, rep(1, nrow(Data10092019)))~ Albuming + NumMets +
LungMet +
        BrainMet + ECOGscore + HepaticMet + Hosp3moconsult + BMI + NumberPalliativeChemo
+
        CodedBreastProstate + AgeTx,  data = Data10092019)

summary(mvcox)
```

© 2021 Wu SY et al. *JAMA Network Open.*

RPA for death

```
#RPA.r
#imports Data10092019met database and reformats variables appropriately
#computes RPA and prints out outcomes data

#appropriately format variables
Data10092019$SexCod <- as.factor(Data10092019$SexCod)
Data10092019$RaceCod <- as.factor(Data10092019$RaceCod)
Data10092019$TxSiteGen <- as.factor(Data10092019$TxSiteGen)
Data10092019$TxSiteSpec <- as.factor(Data10092019$TxSiteSpec)
Data10092019$CodedBreastProstate <- as.factor(Data10092019$CodedBreastProstate)
Data10092019$Hosp3moconsult <- as.factor(Data10092019$Hosp3moconsult)
Data10092019$HepaticMet <- as.factor(Data10092019$HepaticMet)
#Data10092019$ECOGscore <- as.numeric(Data10092019$ECOGscore)
Data10092019$OtherMets <- as.factor(Data10092019$OtherMets)
Data10092019$MetDx <- as.factor(Data10092019$MetDx)
Data10092019$BrainMet <- as.factor(Data10092019$BrainMet)
Data10092019$LungMet <- as.factor(Data10092019$LungMet)
Data10092019$InpatientCoded <- as.factor(Data10092019$InpatientCoded)
Data10092019$NumMets <- as.factor(Data10092019$NumMets)
Data10092019$PtBase <- as.factor(Data10092019$PtBase)
Data10092019$CodIncompleeFx <- as.factor(Data10092019$CodIncompleeFx)

#RPA w StartToDeath
library(rpart)
library(survival)
set.seed(220)
#include all variables to start
osrpa <- rpart(Surv(StartToDeath, rep(1, nrow(Data10092019))) ~ SexCod + RaceCod +
TxSiteGen +
        CodedBreastProstate + Hosp3moconsult + HepaticMet + ECOGscore + OtherMets +
        MetDx + BrainMet + LungMet + InpatientCoded + NumMets + PtBase + AgeTx +
NumberPalliativeChemo + BMI,
        data = Data10092019)
library(rpart.plot)
prp(osrpa) #print pre-parsed

#check to prune the tree
plotcp(osrpa) #print out plot to determine the appropriate complexity parameter
prunedos <- prune(osrpa, 0.014)
prp(prunedos)
```

© 2021 Wu SY et al. *JAMA Network Open.*
